# A Reasonable Diet Promotes Balance of Intestinal Microbiota: Prevention of Precolorectal Cancer

**DOI:** 10.1155/2019/3405278

**Published:** 2019-07-25

**Authors:** Pan Huang, Yi Liu

**Affiliations:** Key Laboratory of Agro-Ecological Processes in Subtropical Regions and Taoyuan Station of Agro-Ecology Research, Institute of Subtropical Agriculture, Chinese Academy of Sciences, Changsha 410125, China

## Abstract

Colorectal cancer (CRC) is a multifactorial disease and the second leading cause of cancer death worldwide. The pathogenesis of colorectal cancer includes genetics, age, chronic inflammation, and lifestyle. Increasing attention has recently been paid to dietary factors. Evidence from epidemiological studies and clinical research suggests that high-fibre diets can significantly reduce the incidence of CRC, whilst the consumption of high-fat diets, high-protein diets, red meat, and processed meat is high-risk factors for tumorigenesis. Fibre is a regulator of intestinal microflora and metabolism and is thus a key dietary component for maintaining intestinal health. Intestinal microbes are closely linked to CRC, with the growth of certain microbiota (such as* Fusobacterium nucleatum*,* Escherichia coli*, or* Bacteroides fragilis*) favouring carcinogenesis, whilst the dominant microbiota population of the intestine, such as* Bacteroidetes, Firmicutes, Actinobacteria*, and* Proteobacteria,* have multiple mechanisms of antitumour activity. Various dietary components have direct effects on the types of intestinal microflora: in the Western diet mode (high-fat, high-protein, and red meat), the proportion of conditional pathogens in the intestinal flora increases, the proportion of commensal bacteria decreases, and the occurrence of colorectal cancer is promoted. Conversely, a high-fibre diet can increase the abundance of* Firmicutes* and reduce the abundance of* Bacteroides* and consequently increase the concentration of short-chain fatty acids (SCFAs) in the intestine, inhibiting the development of CRC. This article reviews the study of the relationship between diet, intestinal microbes, and the promotion or inhibition of CRC and analyses the relevant molecular mechanisms to provide ideas for the prevention and treatment of CRC.

## 1. Introduction

Colorectal cancer (CRC) is one of the world's most common cancers. The incidence of CRC in the United States has ranked in the top three for several consecutive years, with the American Cancer Society estimating that in 2019 CRC will constitute the third most common cause of cancer cases and deaths for both sexes [1].

CRC is not only high in incidence but also high in mortality rate. In the 2018 Global Cancer Survey, CRC mortality ranks second (9.2%) [2], and the age of onset is gradually decreasing [3, 4]. Early CRC is generally asymptomatic, and once symptoms appear the cancer is usually well advanced, so it can only be prevented by regular screening [5]. CRC has many causes, and it is generally believed that genetics, age, environment, lifestyle, and other factors affect its occurrence CRC [6–8]. Among these other factors, dietary patterns have received the most recent attention [9–12].

Different dietary components have different effects on the potential to develop CRC. It is generally believed that a high-fat, high-protein diet including red meat and processed meats promotes the development of CRC, whilst a high-fibre diet inhibits its occurrence [13–17]. High-fat consumption increases the synthesis of intrahepatic bile acids, which are transported to the colon and metabolised by the microbial community into products with tumorigenic activity (secondary bile acids) in the colon [18–20]. Red meat, processed meat, and protein are enzymatically digested by the gut into toxic nitrogen and sulfur-containing substances in the large intestine, which promote CRC [11, 21].

In contrast, soluble fibre is fermented into short-chain fatty acids (SCFAs) by bacteria in the large intestine, and SCFAs including butyrate serve as the main source of energy for colon cells and play an important role in the energy homeostasis of colon tissue [22]. Moreover, a high-fibre intake can increase the number of butyrate-producing bacteria in the gut, such as* Clostridium*,* Anaerostipes*, and* Eubacterium* species [23].

Human studies, preclinical studies, and epidemiological studies have demonstrated a correlation between gut microbiota and CRC [24–28], with the composition of microbes in the intestines or faeces of patients with CRC and precancerous lesions being different from that of healthy control subjects [29, 30]. Consistent data show that the microbiota of patients with CRC is rich in* Fusobacterium nucleatum*,* Bacteroides fragilis,* and* Escherichia coli*, whereas butyrate-producing bacteria such as* Bacteroidetes*,* Firmicutes*,* Echinococcus,* and* Proteobacteria* are depleted in the microbiota of cancer patients [24, 31].

**Figure 1:**
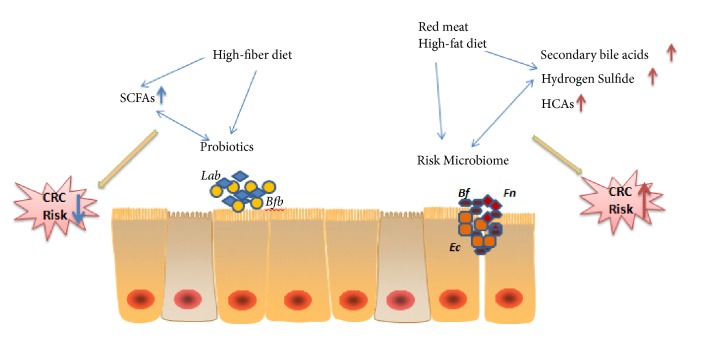
Relationship between diet, gut microbes, and CRC. This figure shows a series of interactions between diet, gut microbiota, and CRC. Soluble fibre is fermented into short-chain fatty acids (SCFAs) by bacteria in the colon. SCFAs are considered to be key metabolites linking gut microbes and a significantly reduced risk of CRC, whilst high-fat diets, high-protein diets, and red meat (a typical Western diet) are metabolised by the gut microbiota into metabolites such as secondary bile acids, heterocyclic amines (HCAs), and hydrogen sulphide, increasing the risk of CRC. At the same time, dietary factors have a regulatory effect on intestinal microbes: long-term consumption of red meat and high-fat diets can increase the proportion of conditional pathogens such as* Fusobacterium nucleatum* (*Fn*),* Escherichia coli* (*Ec*), or* Bacteroides fragilis* (*Bf*) in the gut microbiota, and these bacteria and their metabolites can cause barrier dysfunction, inflammation, and other deleterious changes that increase the risk of CRC. A high-fibre diet can increase the abundance of probiotic bacteria such as* Bifidobacterium* (*Bfb*) and* Lactobacillus* (*Lab*), promote intestinal health, and effectively prevent CRC.

Herein, we provide an overview of the effects of diet on CRC and its possible molecular mechanisms, review the evidence that the intestinal microbiota affects CRC, and discuss potential interactions between diet, the gut microbiota, and CRC (Figure 1).

## 2. Effects of Different Dietary Patterns on the Occurrence of Colorectal Cancer

### 2.1. High-Fat Diet

In 1973, Drasar and Irving first proposed that colorectal cancer and fat are highly correlated [32], which was consistent with the earlier experimental results of Gregor et al. [33] and Wyndr and Shigematsu [34]. Gregor's study highlighted that the mortality of gastric cancer and colorectal cancer was related to animal protein intake, and Wyndr and Shigematsu found that the inhabitants of areas with a high incidence of colorectal cancer usually had a high-fat diet pattern. Related research in this area has since burgeoned, confirming Drasar and Irving's original hypothesis.

We know that fat absorption needs to increase bile flow and that a high-fat diet affects bile metabolism. Data indicate that a high-fat diet increases bile acid concentrations in the faeces [35], and Narisawa et al. [36] reported in 1974 the role of bile acids in the colon promoting cancer development in rats. In 1990, Walter C. Willett et al. [37] conducted used questionnaires to survey and follow up more than 80,000 women of different ages and found that within this population, a large intake of animal fat increased the risk of colon cancer. A study in 2000 confirmed that increased deoxybile acid levels in bile acids were associated with an increased risk of colon cancer, and the transporter gene of bile acid was identified in* Clostridium* sp. strain TO-931 isolated from human faeces [38].

In 2011, researchers conducted experiments on wild-type mice to prove that deoxycholic acid derived from components of a high-fat diet is a colon carcinogen and that some dietary antioxidants can enhance this carcinogenicity [39]. High-fat diets can cause intestinal flora imbalance, increase intestinal permeability and reduce intestinal barrier function [40]. In this way, harmful bacterial products such as lipopolysaccharides (LPS) can enter the intestinal circulation and cause inflammation [41, 42]. Viggiano et al. [43] found that adenosine monophosphate-dependent kinase (AMPK) activation, inflammation and oxidative stress were increased in the hypothalamus of lard-fed rats compared with those fed a control diet, whereas no changes were observed in rats fed fish oil, suggesting that a saturated fat-based diet promotes hypothalamic inflammation and oxidative stress. Studies have also shown that, in an inflammatory environment, a high-fat diet can cause an increase in the concentration of* E. coli*, an increase in the thickness of the mucus layer, an increase in intestinal permeability, upregulation of Nod2 and Tlr5 expression, and promotion of TNF-*α* secretion. These changes lead to the colonisation of the gut by adherent-invasive* Escherichia coli* (AIEC) bacteria, which induce inflammation in the intestinal mucosa and promote the development of CRC [44].

In summary, high-fat diets promote CRC by affecting bile acid metabolism, damaging the integrity of the intestinal barrier and perturbing the intestinal microbiota.

### 2.2. Red Meat

Although academics still dispute whether typical consumption of red meat causes cancer [45], a growing number of convincing studies [21, 46] have shown that red meat and processed meat increase the risk of colon cancer. In 2015, the International Agency for Research on Cancer (IARC) [47] evaluated red meat as “probably carcinogenic to humans” and processed meat as “carcinogenic to humans”, indicating that red meat consumption may be associated with CRC development. Researchers have studied the relationship between colon cancer and meat intake in more than 600 colon cancer cases and 1,000 controls collected from 1996 to 2000 [48] and found that when people eat more high-temperature-cooked red meat, they are more likely to develop colon cancer. This is suggested be at least partly due to the heterocyclic amines (HCAs) produced in this cooking mode, of which 2-amino-3,4,8-trimethylimidazo[4,5-f]quinoxaline (DiMeIQx) is the most common.

Red meat is typically red because it is rich in myoglobin, and haem iron is extremely high in red meat myoglobin [49]. Corpet's study [50] described the dose-response relationship between haem iron levels and colon cancer promotion, and their results in rat experiments suggest that haem iron promotes rectal cancer through direct or indirect effects. In addition, studies have shown that [51] trimethylamine N-oxide (TMAO) in high-fat diet mice can impair glucose tolerance, impede the hepatic insulin-signalling pathway, and lead to inflammation of adipose tissue. Studies by Xu et al. [52] have shown that TMAO is genetically associated with colorectal cancer and that TMAO may be an important intermediate marker linking the metabolism of meat and fat in the diet and gut microbiota to CRC risk.

A large number of previous studies have found that TMAO is produced by gut microbiota metabolism of dietary l-carnitine (a trimethylamine that is present in high levels in red meat) and phosphatidylcholine and is mechanistically linked to the risk of cardiovascular disease (CVD) [53–56]. Koeth et al. [55] showed that the intestinal microbial metabolism of l-carnitine in the diet also produces TMAO, which accelerates atherosclerosis in mice. Wilson Tang et al. proposed that dietary phosphatidylcholine conversion to TMAO depends on the metabolism of intestinal microbiota [53].

In summary, the possible mechanisms of CRC caused by red meat-rich diet primarily involve HCAs generated in the cooking process, haem iron, intestinal microorganisms, and their metabolites, such as TMAO.

### 2.3. High-Protein Diet

Epidemiological studies suggest that high-protein levels in the diet may increase the risk of colorectal cancer [57, 58], but this conclusion remains controversial [59, 60]. Shusuke Toden et al. first studied [61] the association between dietary-resistant starch (RS) and protein and genetic damage of colon cells in rats and obtained results that suggested that increased intake of protein (such as casein) may have deleterious effects on the intestine, whilst fermented complex carbohydrates (such as RS) can counteract these adverse effects in the intestine. Subsequent research by the same group [62] also demonstrated that increased DNA damage caused by high-protein diets (such as cooked red meat or casein) may increase the risk of CRC. Mireille Andriamihaja et al. [63] found that a high-protein diet caused a significant change in the lumen environment of colon cells and the characteristics of these cells, which suggests that a high-protein diet interferes with the metabolism and morphology of colon cells and that it is recommended to consider a high-protein diet cautiously.

Conversely, Sun et al. [64] found that a high-protein diet may actually reduce the risk of cancer. One possible explanation for this finding is that low protein intake may lead to abnormal DNA methylation [65], triggering deactivation of tumour-suppressor genes or related deleterious epigenetic changes.

The small intestine digests and absorbs protein more efficiently than other parts of the intestine (i.e., >95% of the protein that passes through the stomach) [66], so only a small number of proteins that are not completely digested by enzymes enter the large intestine and are microbially hydrolysed [67]. Bacteria such as* Bacillus*,* Streptococcus*,* Propionibacterium*,* Clostridium,* and* Bactericides *can degrade proteins in the large intestine in addition to producing many SCFAs, and the large intestine also metabolises nitrogen and sulfur metabolites such as ammonia, amines, nitrates, nitrites, and hydrogen sulfide, all of which are strong carcinogens or genotoxic substances [11, 68].

In summary, the mechanisms by which a high-protein diet may cause CRC may involve DNA damage and epigenetic changes, interference with the metabolism and morphology of colon cells and toxic metabolites produced by intestinal microorganisms during protein digestion.

### 2.4. High-Fibre Diet

Most studies on dietary fibre and CRC risk have suggested that dietary fibre intake may reduce the risk of CRC [69–71]. In 1971, based on a survey of the prevalence of a high-fibre diet and of CRC in Africa, Burkitt [72] speculated that a high-fibre diet may reduce the incidence of colorectal cancer by swelling the faeces and promoting intestinal peristalsis. Hill [73] also determined that when fibre is not digested in the small intestine, bacteria ferment fibre in the colon, leading to an increase in faecal expansion weight and a shortening of the intestinal transit time, thereby accelerating the excretion of toxic substances.

A high-fibre diet also promotes the growth of beneficial flora in the gut, which binds to bile acids in the intestines and reduces levels of carcinogenic secondary bile acid production [74, 75]. In addition, dietary fibre is enzymatically hydrolysed in the colon to produce SCFAs, such as acetic acid, propionic acid and butyric acid. SCFAs have the function of keeping the intestinal flora stable, inhibiting the proliferation of cancer cells and promoting apoptosis [76, 77]. Butyrate acts as a major source of energy for intestinal epithelial cells and exerts anticancer effects via various pathways, such as upregulation of Bak gene expression and downregulation of Bcl-XL gene expression to induce apoptosis in human colon cancer cells [78]. Butyrate also increases antioxidant activity, thereby inhibiting formation of reactive nitrogen fragments (RONS), an important pathogenic factor in intestinal inflammation and CRC [79, 80], which functions by activating histone deacetylases (HDACs), leading to the proliferation of undifferentiated, highly proliferating colonic adenocarcinoma cells and inhibition of cell differentiation and apoptosis [81].

In summary, the possible mechanisms by which a high-fibre diet can prevent CRC may involve enhancing water-absorbing expansion of faecal matter to promote intestinal movement and accelerate the expulsion of carcinogens, promotion of the growth of beneficial flora to reduce the level of secondary bile acid, and the protective effect of SCFAs generated by fibre-processing by intestinal flora.

## 3. Relationship between Intestinal Microbes and CRC

The human intestine has a large number of microorganisms (78% of the total number of microorganisms in and on the human body) [82–84], and they have a symbiotic relationship with the human host. The microbial population promotes the growth and development of intestinal epithelial cells by participating in the metabolism of the three major nutrients (sugar, protein, fat) in the host to maintain the stability of the alimentary environment [85, 86], and the balance of intestinal microecology is the basis for the normal functioning of other organs [87, 88]. When infection, stress, eating habits, and other factors change, the intestinal microecological balance is perturbed, and the type, quantity, and structure of the intestinal flora changes, leading to an intestinal flora imbalance. This in turn affects the host's health, potentially leading to disease such as CRC, liver cancer, and diabetes [89, 90].

### 3.1. Imbalance of Intestinal Microflora Induces Colorectal Cancer

The interaction between gut microbiota and CRC depends mainly on the balance of microbial populations. Microbial studies have shown that microbial composition is disturbed in colorectal cancer and precancerous lesions [29, 30]. Several studies have shown that* Fusobacterium nucleatum* is closely tied to human CRC.

Kostic et al. [91] described the composition of microflora in colorectal cancer by using the whole genome sequence of nine pairs of tumour group/normal group subjects. Castellari et al. [92] verified the overexpression of the* Fusobacterium* sequence in tumours relative to normal control tissues by quantitative PCR analysis of 99 subjects, observing a positive correlation of overexpression with lymph node metastasis. Related studies suggest that the abundance of* Fusobacterium nucleatum* (*Fn*) in stool samples of colon cancer patients is significantly higher than that in a healthy control group, and an enrichment of* Fn* is also found in colon cancer adenomas [93]. Hashemi Goradel et al. [94] summarised the possible mechanisms of* Fn* action in CRC in a 2018 publication, listing these as immunomodulation, virulence factors, miRNAs, and bacterial metabolism.

*E. coli* promotes the development of intestinal malignancy by inducing local inflammation. Lipopolysaccharides (LPS) produced by Gram-negative bacteria such as* E. coli* increase the expression of Toll-like receptor-4 (TLR4). Activation of TLR4 may promote the occurrence of CRC via Cox-2 expression enhancement and epidermal growth factor receptor (EGFR) signal transduction and other mechanisms [95]. Bacteriocin, a bactericidal protein produced by* Enterobacteriaceae *bacteria, is generally thought to be associated with some intestinal diseases, and the levels of* Enterobacteriaceae* are significantly elevated in patients with CRC [96]. Raisch et al. [88] showed by in vivo experiments with a model of chronic infection of CEACAM6-expressing mice that a B2* E. coli *strain 11G5 was isolatable from colon cancer and persists in the intestine and induces colonic inflammation, epithelial damage and cell proliferation [97].* Clostridium coli* and* E. coli* induce CRC mainly by inducing the inflammatory mode of the tumour microenvironment and producing carcinogenic substances [98, 99].

The study found that the colonic commensal bacteria* Bacteroides fragilis* [100], which is closely related to mucosal colonisation, is associated with acute diarrheal disease, inflammatory bowel disease and CRC [101–103], In particular, high levels of* Bacteroides fragiles *toxin genes (BFT) have been found in advanced CRC [104], and this toxin can stimulate the production of interleukin 17 (IL-17), a proinflammatory cytokine, and thus induce inflammation. In addition, BFT degrades E-cadherin, disrupting epithelial homeostasis and leading to colonic epithelial cell proliferation and possibly CRC [104, 105]. A number of studies have shown that the synergistic effects of various bacterial strains in immune regulation may be involved in the promotion of colon tumorigenesis.

### 3.2. Intestinal Probiotics Inhibit Inflammation and Tumours

Intestinal probiotics can promote the maintenance of normal intestinal microecology and protect the host against CRC by generation of beneficial metabolites, immune tolerance induction and pathogen resistance [89, 106]. The dominant intestinal flora is* Bacteroidetes, Firmicutes, Echinococcus, *and* Proteobacteria*. The most common groups of probiotics are* Lactobacillus *and* Bifidobacterium *[107].* Lactobacillus* can inhibit the expression of tumour-specific proteins and polyamines. Orlando et al. [108] found that the increase of* Lactobacillus rhamnosus* strain GG (*L.* GG) concentration has an effect on the growth and proliferation of HGC-27 (human gastric cancer) and DLD-1 (human colonic cancer) cells and that* L*. GG can significantly reduce synthesis of polyamine metabolites.

Shida et al. [109] tested the interleukin 12 (IL-12) induction ability of 47 strains of* Lactobacillus* present in ten kinds of mouse peritoneal macrophages (IL-12 is a cytokine that plays a key role in activating innate immunity), and it was found that almost all strains belonged to the* Lactobacillus casei *group (*L. casei*,* Lactobacillus rhamnosus*, and* Lactobacillus zeae*) and that* Lactobacillus fermentum* can induce high levels of IL-12. Ma et al. [110] studied the effect of* Bacillus* on tumour growth and found that* Bacillus* inhibits tumour growth by inhibiting ErbB2 and ErbB3, suggesting that* Bacillus* could be a clinical preventive measure for colon cancer. Kim et al. [111] evaluated the anticancer activity and bacterial enzyme inhibition of* Bifidobacterium* sp. SPM0212, which inhibited the proliferation of three human colon cancer cell lines (HT-29, SW480, and Caco-2), and found that* B. adolescentis* SPM0212 also inhibits harmful faecal enzymes.

## 4. Effects of Dietary Ingredients on Intestinal Microbes

Dietary factors have regulatory effects on intestinal symbiotic bacteria as a whole and on bacteria such as* Lactobacillus* and* Clostridium*. For example, long-term consumption of foods high in fat, sugar, protein, red meat, and processed meat can increase the proportion of opportunistic pathogens in intestinal flora and decrease the proportion of symbiotic bacteria [112]. However, a high-fibre diet can increase the abundance of* Firmicutes*, reduce the abundance of* Bacteroides* and increase the SCFA concentration in the intestinal tract, all of which can promote the efficacy of immunotherapy [113].

High-fat diets stimulate the metabolisation of bile acids by microorganisms into carcinogens (such as secondary bile acids) or promote inflammation, increasing the risk of CRC [11, 59]. Bile acids are produced in the liver and are metabolised by microbes in the gut and regulate many metabolic pathways in the host. In addition, bile acids regulate the composition of gut microbes by activating the innate immune genes in the small intestine [114]. Higashimura et al. [115] reported the effects of intestinal dysregulation induced by a high-fat diet in mice, and the use of terminal restriction fragment-length polymorphism (t-rflp) to analyse faecal microbiota showed a decrease in lactic acid bacteria in the HFD group and an increase in the* Clostridium difficile *subgroup XIVa, and HPLC-MS determination of cecal organic acid and serum bile acid content found that secondary bile acid levels had increased. Similarly, another mouse study showed that the relative proportion of faecal F/B (the relative proportion of* Firmicutes *and* Bacteroidetes*) in the high-fat diet group was significantly higher than that in the low-fat diet group [116] and that the F/B ratio was considered to be a good indicator of significant changes in the composition of intestinal microbial community [117].

Earlier studies compared the faecal microbiota in African rural children and that in European children. High-throughput 16S rDNA sequencing and biochemical analysis revealed significant differences in the intestinal flora between the two groups: African children had significant enrichment in* Bacteroides* and reductions in* Firmicutes* and significantly higher expression of SCFAs than EU children [118]. Moreover,* Enterobacteriaceae* levels were significantly lower than in EU children. Further analysis showed that the* Bacteroides* levels in the intestine were associated with the consumption of animal protein and saturated fat level, supporting the contention that meat consumption in the Western diet is driving the intestinal phenotype. The researchers also noted that changes in the proportion of intestinal microbes may be related to a decrease in dietary fibre content and that the lack of colonic butyrate enhances the carcinogenic potential of meat and fat-stimulated metabolites [62, 119, 120].

Dietary fibre refers to the edible part of a plant or its extract or similar carbohydrate that is not absorbed and use in the small intestine, but is utilised by the microbial population that is resident after partial or complete fermentation in the large intestine [17]. Foods high in dietary fibre reduce the bacterial abundance of* Bacteroides* and* Helicobacter* species and enrich the levels of* Ruminococci*, which degrade dietary fibre and produce short-chain fatty acids [74].

O'Keefe et al. [121] conducted a 2-week dietary intervention in African Americans and rural occupants in South Africa. South African aborigines switched from a high-fibre diet to a low-fibre diet, whilst the African Americans switched from a low-fibre diet to a high-fibre diet. This resulted in an altered metabolism and metabolome in both groups, including a change in levels of SCFAs such as butyrate. Koropatkin et al. [122] found that adjusting the intake of RS or nonstarch polysaccharides in the diet can change the distribution of bacteria such as* Ruminococcus* and* Eubacterium rectale,* and analysis of faecal samples in vitro confirmed that these bacteria could selectively metabolise insoluble carbohydrates and metabolise polysaccharides by fermentation to produce SCFAs.

A mixture of inulin and oligofructose supplements in human diet experiments can stimulate* Bifidobacteria*, especially the proliferation of* Bifidobacterium adolescentis* and* Faecalibacterium prausnitzii *[123]. In addition, the SCFAs produced by intestinal flora metabolism can activate G-protein coupled receptors and regulatory T cells, increasing mucosal immune tolerance [124, 125]. Various dietary components can fuel different intestinal microflora and thus affect the metabolic function of the microorganisms, thereby regulating the hosts' physiological functions.

## 5. Conclusions and Outlook

Diet is closely related to CRC pathogenesis: a high-fat diet increases bile acid concentration and damages the intestinal barrier and its function. Red meat and a high-protein diet favour the formation of nitrogen and sulfur metabolites, which are beneficial to the growth of proinflammatory intestinal bacteria. However, a high-fibre diet stimulates production of protective or anti-inflammatory components, such as SCFAs, that protect the gut.

Dietary changes can significantly affect gut health, and people with different dietary patterns will have different gut microflora, and dietary components have been convincingly demonstrated to shape the gut microbiota throughout life. The microbiota can also affect the health of the host intestinal epithelial cells, and evidence even shows that the gut microbiota regulate the host's satiety and brain function (denoted the visceral-cerebral axis) [126, 127].

A series of interactions exist between dietary factors, gut microbiota and CRC. Excessive intake of saturated fats and processed meat lead to an increase in harmful bacteria such as* Fn* and* Ec*, which changes the permeability of intestinal mucosa, induces inflammation and promotes the development of CRC. People with a high-fibre diet therefore have a healthier intestinal microecology. Numerous studies have shown that dietary intervention and regulation of intestinal flora are the most cost-effective strategies for the prevention of CRC, and we strongly recommend a balanced diet rich in fibre.

## References

[1] Siegel R. L., Miller K. D., Jemal A. (2019). Cancer statistics, 2019. *CA: A Cancer Journal for Clinicians*.

[2] Bray F., Ferlay J., Soerjomataram I. (2018). Global cancer statistics 2018: GLOBOCAN estimates of incidence and mortality worldwide for 36 cancers in 185 countries. *CA: A Cancer Journal for Clinicians*.

[3] Weinberg B. A., Marshall J. L. (2019). Colon cancer in young adults: trends and their implications. *Current Oncology Reports*.

[4] Siegel R. L., Fedewa S. A., Anderson W. F. (2017). Colorectal cancer incidence patterns in the United States, 1974–2013. *JNCI: Journal of the National Cancer Institute*.

[5] Block K. I., Block P. B., Gyllenhaal C. (2018). Integrative treatment for colorectal cancer: a comprehensive approach. *The Journal of Alternative and Complementary Medicine*.

[6] Lasko C. M., Bird R. P. (1995). Modulation of aberrant crypt foci by dietary fat and caloric restriction: the effects of delayed intervention. *Cancer Epidemiology, Biomarkers & Prevention: A Publication of The American Association for Cancer Research, Cosponsored by the American Society of Preventive Oncology*.

[7] Key T. J., Allen N. E., Spencer E. A., Travis R. C. (2002). The effect of diet on risk of cancer. *The Lancet*.

[8] Song M., Chan A. T. (2019). Environmental factors, gut microbiota, and colorectal cancer prevention. *Clinical Gastroenterology and Hepatology*.

[9] Imperiale T. F., Abhyankar P. R., Stump T. E., Emmett T. W. (2018). Prevalence of advanced, precancerous colorectal neoplasms in black and white populations: a systematic review and meta-analysis. *Gastroenterology*.

[10] Giovannucci E., Goldin B. (1997). The role of fat, fatty acids, and total energy intake in the etiology of human colon cancer. *American Journal of Clinical Nutrition*.

[11] O'Keefe S. J. D. (2016). Diet, microorganisms and their metabolites, and colon cancer. *Nature Reviews Gastroenterology & Hepatology*.

[12] Niederreiter L., Adolph T. E., Tilg H. (2018). Food, microbiome and colorectal cancer. *Digestive and Liver Disease*.

[13] Karunanithi S., Levi L. (2018). High-fat diet and colorectal cancer: myths and facts. *Future Oncology*.

[14] Newmark H., Yang K., Lipkin M. (2001). A Western-style diet induces benign and malignant neoplasms in the colon of normal C57Bl/6 mice. *Carcinogenesis*.

[15] Beyaz S., Mana M. D., Roper J. (2016). High-fat diet enhances stemness and tumorigenicity of intestinal progenitors. *Nature*.

[16] Nyström M., Mutanen M. (2009). Diet and epigenetics in colon cancer. *World Journal of Gastroenterology*.

[17] Bultman S. J. (2017). Interplay between diet, gut microbiota, epigenetic events, and colorectal cancer. *Molecular Nutrition & Food Research*.

[18] Moreira A. P. B., Texeira T. F. S., Ferreira A. B., Do Carmo Gouveia Peluzio M., De Cássia Gonçalves Alfenas R. (2012). Influence of a high-fat diet on gut microbiota, intestinal permeability and metabolic endotoxaemia. *British Journal of Nutrition*.

[19] Ridlon J. M., Kang D.-J., Hylemon P. B. (2006). Bile salt biotransformations by human intestinal bacteria. *Journal of Lipid Research*.

[20] Hofmann A. F. (1999). The continuing importance of bile acids in liver and intestinal disease. *JAMA Internal Medicine*.

[21] Norat T., Lukanova A., Ferrari P., Riboli E. (2002). Meat consumption and colorectal cancer risk: Dose-response meta-analysis of epidemiological studies. *International Journal of Cancer*.

[22] Donohoe D. R., Garge N., Zhang X. (2011). The microbiome and butyrate regulate energy metabolism and autophagy in the mammalian colon. *Cell Metabolism*.

[23] Chen H., Yu Y., Wang J. (2013). Decreased dietary fiber intake and structural alteration of gut microbiota in patients with advanced colorectal adenoma. *American Journal of Clinical Nutrition*.

[24] Sears C. L., Garrett W. S. (2014). Microbes, microbiota, and colon cancer. *Cell Host & Microbe*.

[25] Flemer B., Lynch D. B., Brown J. M. R. (2017). Tumour-associated and non-tumour-associated microbiota in colorectal cancer. *Gut*.

[26] Zeller G., Tap J., Voigt A. Y. (2015). Potential of fecal microbiota for early-stage detection of colorectal cancer. *Molecular Systems Biology*.

[27] Ahn J., Sinha R., Pei Z. (2013). Human gut microbiome and risk for colorectal cancer. *JNCI Journal of the National Cancer Institute*.

[28] Sobhani I., Tap J., Roudot-Thoraval F. (2011). Microbial dysbiosis in colorectal cancer (CRC) patients. *Plos One*.

[29] Nakatsu G., Li X., Zhou H. (2015). Gut mucosal microbiome across stages of colorectal carcinogenesis. *Nature Communications*.

[30] Feng Q., Liang S., Jia H. (2015). Gut microbiome development along the colorectal adenoma-carcinoma sequence. *Nature Communications*.

[31] Tilg H., Adolph T. E., Gerner R. R., Moschen A. R. (2018). The intestinal microbiota in colorectal cancer. *Cancer Cell*.

[32] Drasar B. S., Irving D. (1973). Environmental factors and cancer of the colon and breast. *British Journal of Cancer*.

[33] Gregor O., Toman R., Prusova F. (1969). Gastrointestinal cancer and nutrition. *Gut*.

[34] Wynder E. L., Shigematsu T. (1967). Environmental factors of cancer of the colon and rectum. *Cancer*.

[35] Cummings J. H., Wiggins H. S., Jenkins D. J. A. (1978). Influence of diets high and low in animal fat on bowel habit, gastrointestinal transit time, fecal microflora, bile acid, and fat excretion. *The Journal of Clinical Investigation*.

[36] Narisawa T., Magadia N. E., Weisburger J. H., Wynder E. L. (1974). Promoting effect of bile acids on colon carcinogenesis after intrarectal instillation of n-methyl-n′ nitro-n-nitrosoguanidine in rats2. *JNCI: Journal of the National Cancer Institute*.

[37] Willett W. C., Stampfer M. J., Colditz G. A., Rosner B. A., Speizer F. E. (1990). Relation of meat, fat, and fiber intake to the risk of colon cancer in a prospective study among women. *The New England Journal of Medicine*.

[38] Wells J. E., Hylemon P. B. (2000). Identification and characterization of a bile acid 7*α*-dehydroxylation operon in Clostridium sp. strain TO-931, a highly active 7*α*-dehydroxylating strain isolated from human feces. *Applied and Environmental Microbiology*.

[39] Bernstein C., Holubec H., Bhattacharyya A. K. (2011). Carcinogenicity of deoxycholate, a secondary bile acid. *Archives of Toxicology*.

[40] Schulz M. D., Atay Ç., Heringer J. (2014). High-fat-diet-mediated dysbiosis promotes intestinal carcinogenesis independently of obesity. *Nature*.

[41] Ghoshal S., Witta J., Zhong J., de Villiers W., Eckhardt E. (2009). Chylomicrons promote intestinal absorption of lipopolysaccharides. *Journal of Lipid Research*.

[42] Kvietys P. R., Specian R. D., Grisham M. B., Tso P. (1992). Jejunal mucosal injury and restitution: role of hydrolytic products of food digestion. *Jpen Journal of Parenteral & Enteral Nutrition*.

[43] Viggiano E., Mollica M. P., Lionetti L. (2016). Effects of an high-fat diet enriched in lard or in fish oil on the hypothalamic amp-activated protein kinase and inflammatory mediators. *Frontiers in Cellular Neuroscience*.

[44] Martinez-Medina M., Denizot J., Dreux N. (2014). Western diet induces dysbiosis with increased *E. coli* in CEABAC10 mice, alters host barrier function favouring AIEC colonisation. *Gut*.

[45] Kruger C., Zhou Y. (2018). Red meat and colon cancer: A review of mechanistic evidence for heme in the context of risk assessment methodology. *Food and Chemical Toxicology*.

[46] Ognjanovic S., Yamamoto J., Maskarinec G., Marchand L. L. (2006). NAT2, meat consumption and colorectal cancer incidence: An ecological study among 27 countries. *Cancer Causes & Control*.

[47] Bouvard V., Loomis D., Guyton K. Z. (2015). Carcinogenicity of consumption of red and processed meat. *The Lancet Oncology*.

[48] Butler L. M. (2003). Heterocyclic amines, meat intake, and association with colon cancer in a population-based study. *American Journal of Epidemiology*.

[49] Sasso A., Latella G. (2019). Role of heme iron in the association between red meat consumption and colorectal cancer. *Nutrition and Cancer*.

[50] Corpet D. E. (2011). Red meat and colon cancer: should we become vegetarians, or can we make meat safer?. *Meat Science*.

[51] Gao X., Liu X., Xu J., Xue C., Xue Y., Wang Y. (2014). Dietary trimethylamine N-oxide exacerbates impaired glucose tolerance in mice fed a high fat diet. *Journal of Bioscience and Bioengineering*.

[52] Xu R., Wang Q., Li L. (2015). A genome-wide systems analysis reveals strong link between colorectal cancer and trimethylamine N-oxide (TMAO), a gut microbial metabolite of dietary meat and fat. *BMC Genomics*.

[53] Tang W. H. W., Wang Z., Levison B. S. (2013). Intestinal microbial metabolism of phosphatidylcholine and cardiovascular risk. *The New England Journal of Medicine*.

[54] Wang Z., Klipfell E., Bennett B. J. (2011). Gut flora metabolism of phosphatidylcholine promotes cardiovascular disease. *Nature*.

[55] Koeth R. A., Wang Z., Levison B. S. (2013). Intestinal microbiota metabolism of L-carnitine, a nutrient in red meat, promotes atherosclerosis. *Nature Medicine*.

[56] Liu G., Yan W., Ding S. (2018). Effects of IRW and IQW on oxidative stress and gut microbiota in dextran sodium sulfate-induced colitis. *Cellular Physiology and Biochemistry*.

[57] Cassidy A., Bingham S., Cummings J. (1994). Starch intake and colorectal cancer risk: an international comparison. *British Journal of Cancer*.

[58] Tayyem R., Bawadi H., Shehadah I. (2015). Macro- and micronutrients consumption and the risk for colorectal cancer among jordanians. *Nutrients*.

[59] Yang J., Yu J. (2018). The association of diet, gut microbiota and colorectal cancer: what we eat may imply what we get. *Protein & Cell*.

[60] Lai R., Bian Z., Lin H., Ren J., Zhou H., Guo H. (2017). The association between dietary protein intake and colorectal cancer risk: a meta-analysis. *World Journal of Surgical Oncology*.

[61] Shusuke T., Bird A. R., Topping D. L., Conlon M. A. (2005). Resistant starch attenuates colonic DNA damage induced by higher dietary protein in rats. *Nutrition & Cancer-An International Journal*.

[62] Toden S., Bird A. R., Topping D. L., Conlon M. A. (2006). Resistant starch prevents colonic DNA damage induced by high dietary cooked red meat or casein in rats. *Cancer Biology & Therapy*.

[63] Andriamihaja M., Davila A., Eklou-Lawson M. (2010). Colon luminal content and epithelial cell morphology are markedly modified in rats fed with a high-protein diet. *American Journal of Physiology-Gastrointestinal and Liver Physiology*.

[64] Sun Z., Liu L., Wang P. P. (2012). Association of total energy intake and macronutrient consumption with colorectal cancer risk: results from a large population-based case-control study in Newfoundland and Labrador and Ontario, Canada. *Nutrition Journal *.

[65] Yamaji T., Iwasaki M., Sasazuki S., Sakamoto H., Yoshida T., Tsugane S. (2009). Methionine synthase A2756G polymorphism interacts with alcohol and folate intake to influence the risk of colorectal adenoma. *Cancer Epidemiology, Biomarkers & Prevention*.

[66] Silvester K. R., Cummings J. H. (1995). Does digestibility of meat protein help explain large bowel cancer risk?. *Nutrition and Cancer*.

[67] Macfarlane G., Gibson G., Cummings J. (1992). Comparison of fermentation reactions in different regions of the human colon. *Journal of Applied Bacteriology*.

[68] Windey K., de Preter V., Verbeke K. (2012). Relevance of protein fermentation to gut health. *Molecular Nutrition & Food Research*.

[69] Maćkowiak K., Torlińska-Walkowiak N., Torlińska B. (2016). Dietary fibre as an important constituent of the diet. *Postepy Higieny i Medycyny Doswiadczalnej*.

[70] Wakai K., Hirose K., Matsuo K. (2006). Dietary risk factors for colon and rectal cancers: a comparative case-control study. *Journal of Epidemiology*.

[71] Bingham S. A., Day N. E., Luben R. (2003). Dietary fibre in food and protection against colorectal cancer in the European Prospective Investigation into Cancer and Nutrition (EPIC): an observational study. *The Lancet*.

[72] Burkitt D. P. (1971). Some neglected leads to cancer causation. *JNCI: Journal of the National Cancer Institute*.

[73] Hill M. J. (1998). Cereals, cereal fibre and colorectal cancer risk. *European Journal of Cancer Prevention*.

[74] Requena T., Martinez-Cuesta M. C., Pelaez C. (2018). Diet and microbiota linked in health and disease. *Food & Function*.

[75] Wakai K., Date C., Fukui M. (2007). Dietary fiber and risk of colorectal cancer in the Japan collaborative cohort study. *Cancer Epidemiology, Biomarkers & Prevention*.

[76] Lipkin M., Reddy B., Newmark H., Lamprecht S. A. (1999). Dietary factors in human colorectal cancer. *Annual Review of Nutrition*.

[77] Lim C. C., Ferguson L. R., Tannock G. W. (2005). Dietary fibres as “prebiotics”: implications for colorectal cancer. *Molecular Nutrition & Food Research*.

[78] Menzel T., Schauber J., Kreth F. (2002). Butyrate and aspirin in combination have an enhanced effect on apoptosis in human colorectal cancer cells. *European Journal of Cancer Prevention*.

[79] Wang L. S., Huang Y. W., Stoner G. D., Lechner J. F. (2012). Gene-diet interactions on colorectal cancer risk. *Current Nutrition Reports*.

[80] Ma Y., Ding S., Liu G. (2019). Egg protein transferrin-derived peptides IRW and IQW regulate citrobacter rodentium-induced, inflammation-related microbial and metabolomic profiles. *Frontiers in Microbiology*.

[81] Comalada M., Bailón E., De Haro O. (2006). The effects of short-chain fatty acids on colon epithelial proliferation and survival depend on the cellular phenotype. *Journal of Cancer Research and Clinical Oncology*.

[82] Tlaskalová-Hogenová H., Tpánková R., Kozáková H. (2011). The role of gut microbiota (commensal bacteria) and the mucosal barrier in the pathogenesis of inflammatory and autoimmune diseases and cancer: Contribution of germ-free and gnotobiotic animal models of human diseases. *Cellular & Molecular Immunology*.

[83] Wang K., Karin M. (2014). Common flora and intestine. *Cellular Logistics*.

[84] Floch M. H. (2011). Intestinal microecology in health and wellness. *Journal of Clinical Gastroenterology*.

[85] Power S. E., O'Toole P. W., Stanton C., Ross R. P., Fitzgerald G. F. (2014). Intestinal microbiota, diet and health. *British Journal of Nutrition*.

[86] Walsh C. J., Guinane C. M., O'Toole P. W., Cotter P. D. (2014). Beneficial modulation of the gut microbiota. *FEBS Letters*.

[87] Ellmerich S., Scholler M., Duranton B. (2000). Promotion of intestinal carcinogenesis by Streptococcus bovis. *Carcinogenesis*.

[88] Raisch J., Buc E., Bonnet M. (2014). Colon cancer-associated B2 Escherichia coli colonize gut mucosa and promote cell proliferation. *World Journal of Gastroenterology*.

[89] Zhu Y., Michelle Luo T., Jobin C., Young H. A. (2011). Gut microbiota and probiotics in colon tumorigenesis. *Cancer Letters*.

[90] Zackular J. P., Baxter N. T., Iverson K. D. (2013). The gut microbiome modulates colon tumorigenesis. *mBio*.

[91] Kostic A. D., Gevers D., Pedamallu C. S. (2012). Genomic analysis identifies association of Fusobacterium with colorectal carcinoma. *Genome Research*.

[92] Castellarin M., Warren R. L., Freeman J. D. (2012). Fusobacterium nucleatum infection is prevalent in human colorectal carcinoma. *Genome Research*.

[93] Flanagan L., Schmid J., Ebert M. (2014). Fusobacterium nucleatum associates with stages of colorectal neoplasia development, colorectal cancer and disease outcome. *European Journal of Clinical Microbiology & Infectious Diseases*.

[94] Hashemi Goradel N., Heidarzadeh S., Jahangiri S. (2018). *Fusobacterium nucleatum* and colorectal cancer: A mechanistic overview. *Journal of Cellular Physiology*.

[95] Fukata M., Chen A., Vamadevan A. S. (2007). Toll-like receptor-4 promotes the development of colitis-associated colorectal tumors. *Gastroenterology*.

[96] Kohoutova D., Smajs D., Moravkova P. (2014). Escherichia colistrains of phylogenetic group B2 and D and bacteriocin production are associated with advanced colorectal neoplasia. *BMC Infectious Diseases*.

[97] Ding S., Liu G., Jiang H., Fang J. (2019). MicroRNA determines the fate of intestinal epithelial cell differentiation and regulates intestinal diseases. *Current Protein & Peptide Science*.

[98] Arthur J. C., Perez-Chanona E., Mühlbauer M. (2012). Intestinal inflammation targets cancer-inducing activity of the microbiota. *Science*.

[99] Kostic A., Chun E., Robertson L. (2013). Fusobacterium nucleatum potentiates intestinal tumorigenesis and modulates the tumor-immune microenvironment. *Cell Host & Microbe*.

[100] Huang J. Y., Lee S. M., Mazmanian S. K. (2011). The human commensal Bacteroides fragilis binds intestinal mucin. *Anaerobe*.

[101] Toprak N. U., Yagci A., Gulluoglu B. M. (2006). A possible role of Bacteroides fragilis enterotoxin in the aetiology of colorectal cancer. *Clinical Microbiology and Infection*.

[102] Basset C., Holton J., Bazeos A., Vaira D., Bloom S. (2004). Are helicobacter species and enterotoxigenic bacteroides fragilis involved in inflammatory bowel disease?. *Digestive Diseases and Sciences*.

[103] Sears C. L. (2009). Enterotoxigenic *Bacteroides fragilis*: a rogue among symbiotes. *Clinical Microbiology Reviews*.

[104] Boleij A., Hechenbleikner E. M., Goodwin A. C. (2015). The bacteroides fragilis toxin gene is prevalent in the colon mucosa of colorectal cancer patients. *Clinical Infectious Diseases*.

[105] Sears C. L., Geis A. L., Housseau F. (2014). Bacteroides fragilis subverts mucosal biology: from symbiont to colon carcinogenesis. *The Journal of Clinical Investigation*.

[106] Sivan A., Corrales L., Hubert N. (2015). Commensal Bifidobacterium promotes antitumor immunity and facilitates anti-PD-L1 efficacy. *Science*.

[107] Eslami M., Yousefi B., Kokhaei P. (2019). Importance of probiotics in the prevention and treatment of colorectal cancer. *Journal of Cellular Physiology*.

[108] Orlando A., Messa C., Linsalata M., Cavallini A., Russo F. (2009). Effects of Lactobacillus rhamnosus GG on proliferation and polyamine metabolism in HGC-27 human gastric and DLD-1 colonic cancer cell lines. *Immunopharmacology and Immunotoxicology*.

[109] Shida K., Kiyoshima-Shibata J., Nagaoka M., Watanabe K., Nanno M. (2006). Induction of interleukin-12 by Lactobacillus strains having a rigid cell wall resistant to intracellular digestion. *Journal of Dairy Science*.

[110] Ma E. L., Choi Y. J., Choi J., Pothoulakis C., Rhee S. H., Im E. (2010). The anticancer effect of probiotic Bacillus polyfermenticus on human colon cancer cells is mediated through ErbB2 and ErbB3 inhibition. *International Journal of Cancer*.

[111] Kim Y., Lee D., Kim D. (2008). Inhibition of proliferation in colon cancer cell lines and harmful enzyme activity of colon bacteria by Bifidobacterium adolescentis SPM0212. *Archives of Pharmacal Research*.

[112] Agus A., Denizot J., Thévenot J. (2016). Western diet induces a shift in microbiota composition enhancing susceptibility to Adherent-Invasive *E. coli* infection and intestinal inflammation. *Scientific Reports*.

[113] Zou J., Chassaing B., Singh V. (2018). Fiber-mediated nourishment of gut microbiota protects against diet-induced obesity by restoring IL-22-mediated colonic health. *Cell Host & Microbe*.

[114] Wahlström A., Sayin S. I., Marschall H.-U., Bäckhed F. (2016). Intestinal crosstalk between bile acids and microbiota and its impact on host metabolism. *Cell Metabolism*.

[115] Higashimura Y., Naito Y., Takagi T. (2016). Protective effect of agaro-oligosaccharides on gut dysbiosis and colon tumorigenesis in high-fat diet-fed mice. *American Journal of Physiology-Gastrointestinal and Liver Physiology*.

[116] Taira T., Yamaguchi S., Takahashi A. (2015). Dietary polyphenols increase fecal mucin and immunoglobulin A and ameliorate the disturbance in gut microbiota caused by a high fat diet. *Journal of Clinical Biochemistry and Nutrition*.

[117] Guo X., Xia X., Tang R., Zhou J., Zhao H., Wang K. (2008). Development of a real-time PCR method for Firmicutes and Bacteroidetes in faeces and its application to quantify intestinal population of obese and lean pigs. *Letters in Applied Microbiology*.

[118] de Filippo C., Cavalieri D., di Paola M. (2010). Impact of diet in shaping gut microbiota revealed by a comparative study in children from Europe and rural Africa. *Proceedings of the National Acadamy of Sciences of the United States of America*.

[119] van Munster I. P., Tangerman A., Nagengast F. M. (1994). Effect of resistant starch on colonic fermentation, bile acid metabolism, and mucosal proliferation. *Digestive Diseases and Sciences*.

[120] De Boever P., Wouters R., Verschaeve L., Berckmans P., Schoeters G., Verstraete W. (2000). Protective effect of the bile salt hydrolase-active Lactobacillus renteri against bile salt cytotoxicity. *Applied Microbiology and Biotechnology*.

[121] O'Keefe S. J., Li J. V., Lahti L. (2015). Fat, fibre and cancer risk in African Americans and rural Africans. *Nature Communications*.

[122] Koropatkin N. M., Cameron E. A., Martens E. C. (2012). How glycan metabolism shapes the human gut microbiota. *Nature Reviews Microbiology*.

[123] Ramirez-Farias C., Slezak K., Fuller Z., Duncan A., Holtrop G., Louis P. (2009). Effect of inulin on the human gut microbiota: stimulation of Bifidobacterium adolescentis and Faecalibacterium prausnitzii. *British Journal of Nutrition*.

[124] Tremaroli V., Bäckhed F. (2012). Functional interactions between the gut microbiota and host metabolism. *Nature*.

[125] Smith P. M., Howitt M. R., Panikov N. (2013). The microbial metabolites, short-chain fatty acids, regulate colonic T reg cell homeostasis. *Science*.

[126] Fetissov S. O. (2017). Role of the gut microbiota in host appetite control: bacterial growth to animal feeding behaviour. *Nature Reviews Endocrinology*.

[127] Burcelin R. (2017). When gut fermentation controls satiety: A PYY story. *Molecular Metabolism*.

